# Crystal structure and Hirshfeld surface analysis of (*E*)-2-(2,4,6-tri­methyl­benzyl­idene)-3,4-di­hydro­naphthalen-1(2*H*)-one

**DOI:** 10.1107/S2056989019006182

**Published:** 2019-05-10

**Authors:** Cemile Baydere, Merve Taşçı, Necmi Dege, Mustafa Arslan, Yusuf Atalay, Irina A. Golenya

**Affiliations:** a Department of Physics, Faculty of Arts and Sciences, Ondokuz Mayıs University, 55139-Samsun, Turkey; b Sakarya University, Faculty of Arts & Sciences, Chemistry Department, Sakarya, Turkey; c Taras Shevchenko National University of Kyiv, Department of Chemistry, 64, Vladimirska Str., 01601, Kiev, Ukraine

**Keywords:** chalcone, crystal structure, hydrogen bond, Hirshfeld surface analysis

## Abstract

A novel chalcone, C_20_H_20_O, derived from benzyl­idene­tetra­lone, was synthesized *via* Claissen–Schmidt condensation between tetra­lone and 2,4,6-tri­methyl­benzaldehyde. In the crystal, mol­ecules are linked by C—H⋯O hydrogen bonds, producing *R*_2_^2^(20) and *R*_2_^4^(12) ring motifs. In addition, weak C—H⋯π and π-stacking inter­actions are observed. The inter­molecular inter­actions were investigated using Hirshfeld surface analysis and two-dimensional fingerprint plots, revealing that the most important contributions for the crystal packing are from H⋯H (66.0%), H⋯C/ C⋯H (22.3%), H⋯O/O⋯H (9.3%), and C⋯C (2.4%) inter­actions. Shape-index plots show π–π stacking inter­actions and the curvedness plots show flat surface patches characteristic of planar stacking.

## Abstract 2

In the title compound, C—H⋯O hydrogen bonds and weak C—H⋯π inter­actions link adjacent mol­ecules into a three-dimensional supra­molecular network.

## Chemical context   

Chalcone (systematic name 1,3-diphenyl-2-propene-1-one) is an aromatic ketone that represents the central core for various derivatives with inter­esting properties, known as chalcones (Kostanecki & Tambor, 1899 ▸). For example, chalcones are found in fruits, vegetables, spices, tea or soy, and find applications as pharmaceuticals (Di Carlo *et al.*, 1999 ▸). Chalcones are also major inter­mediates in the synthesis of natural products and are widely used in synthetic and pharmaceutical chemistry (Dhar, 1981 ▸; Ansari *et al.*, 2005 ▸) because they have anti­tumor (Modzelewska *et al.*, 2006 ▸), anti­fungal (López *et al.*, 2001 ▸), anti-inflammatory (Lee *et al.*, 2006 ▸), anti-bacterial (Batovska *et al.*, 2009 ▸) or anti­tubercular properties (Lin *et al.*, 2002 ▸). In general, chalcones consist of two aromatic rings that are linked by a three-carbon α,β-unsaturated carbonyl system, leading to a completely delocalized π-electron system. Recently, chalcones have also been used in the field of materials science as non-linear optical devices (Raghavendra *et al.*, 2017 ▸). As part of our studies in this area, we report herein the synthesis, crystal structure and Hirshfeld surface analysis of a new chalcone.
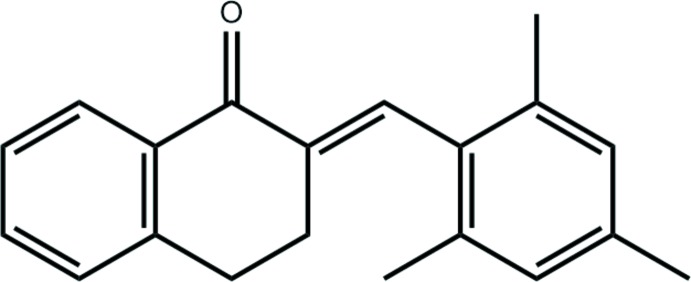


## Structural commentary   

**Figure 1:**
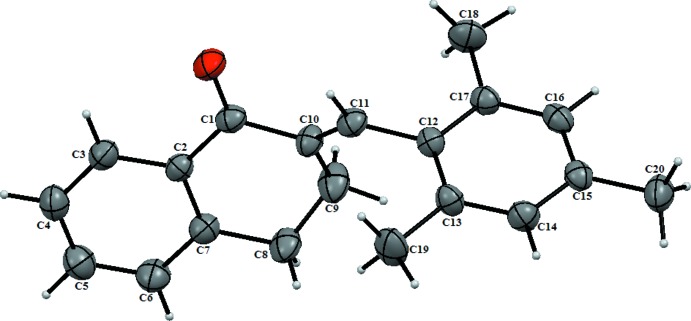
The mol­ecular structure of the title compound, with the atom labelling. Displacement ellipsoids are drawn at the 50% probability level.

In the title mol­ecule (Fig. 1 ▸), the cyclo­hexa­none ring (C1/C2,C7/C8,C9/C10) has an envelope conformation with the flap atom C9 deviating by 0.280 (3) Å from the least-squares plane through the ring. The cyclo­hexa­none ring is nearly co-planar with the benzene ring (C2–C7) being fused at a dihedral angle of 4.70 (18)°, but is inclined to the other benzene ring (C12–C17) by 74.95 (13)°. Torsion angles involving the methyl­ene group C10=C11 are 83.3 (5)° (C17—C12—C11—C10), 129.8 (4)° (C11—C10—C9—C8) and 27.7 (6)° (O1—C1—C10—C11).

## Supra­molecular features   

**Figure 2:**
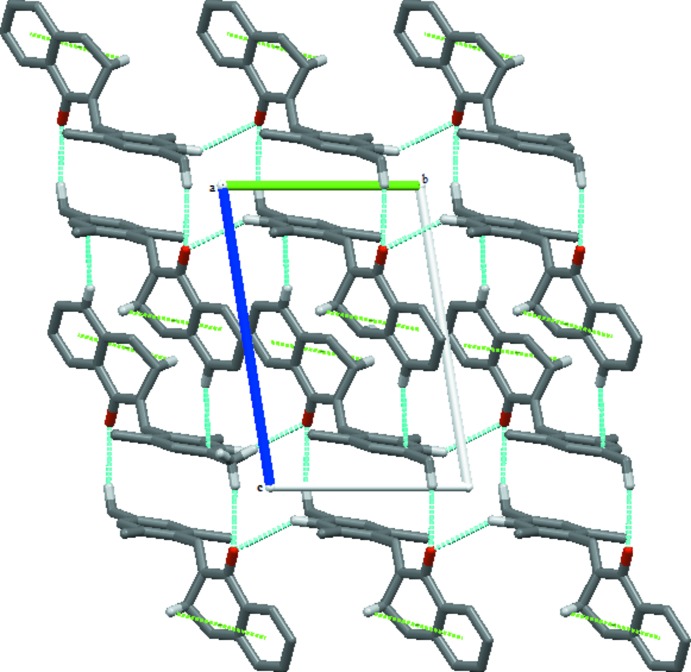
A view along the *a* axis of the title structure. Blue dashed lines denote the C—H⋯O hydrogen bonds which form 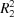
(20) and 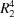
(12) ring motifs. C—H⋯π inter­actions are shown as green dashes lines.

The main inter­molecular inter­actions in the crystal structure of the title compound are of type C—H⋯O, C—H⋯π (Table 1 ▸) and π–π. Inter­actions between a methyl group and the carbonyl O atom (C20—H20*C*⋯O1^ii^) as well as between an aromatic H atom and the carbonyl atom (C16—H16⋯O1^i^) lead to 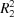
(20) and 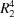
(12) motifs (Fig. 2 ▸), linking adjacent mol­ecules parallel to (001) (Table 2 ▸, Fig. 2 ▸). A weak C9—H9*A*⋯*Cg*2^iii^ (*Cg*2 is the centroid of the C2–C7 benzene ring) inter­action is also present (Fig. 2 ▸), along with weak aromatic π-stacking inter­actions [*Cg*2⋯*Cg*2(−2 − *x*, −*y*, −1 − *z*) = 3.887 (3) Å] that consolidate the three-dimensional packing.

## Database survey   

A search of the Cambridge Structural Database (CSD, version 5.40, update November 2018; Groom *et al.*, 2016 ▸) using (*E*)-2-(4-methyl­benzyl­idene)-3,4-di­hydro­naphthalen-1(2*H*)-one as the main skeleton revealed the presence of four structures containing the chalcone moiety with different substituents that are similar to the title compound: (*E*)-4-[(1-oxo-3,4-di­hydro­naphthalen-2(1*H*)-yl­idene)meth­yl]benzo­nitrile (QEVMAI; Baddeley *et al.*, 2017 ▸); (*E*)-4-[(5-meth­oxy-1-oxo-3,4-di­hydro­naphthalen-2(1*H*)-yl­idene)meth­yl]benzo­nitrile (QEVMEM; Baddeley *et al.*, 2017 ▸); (*E*)-4-[(6-meth­oxy-1-oxo-3,4-di­hydro­naphthalen-2(1*H*)-yl­idene)meth­yl]benzo­nitrile (QEVMIQ; Baddeley *et al.*, 2017 ▸); 1′-(4-bromo­phen­yl)-4′-{4-[(1-oxo-3,4-di­hydro­naphthalen-2(1*H*)-yl­idene) meth­yl]phen­yl}-3′′,4′′-di­hydro-1′′*H*,2*H*-di­spiro­(ace­naphthyl­ene-1,2′-pyrrolidine-3′,2′′-naphthalene)-1′′,2-dione (VUZXOE; Saravanan *et al.*, 2010 ▸). QEVMAI and VUZXOE both crystallize in space group *P* 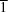, while QEVMEM and QEVMIQ crystallize in space group *P*2_1_/*c*. In the structures of QEVMAI, QEVMEM and QEVMIQ, the dihedral angles between the phenyl groups are 45.66 (5), 55.06 (7) and 69.78 (5)°, respectively. In the structure of VUZXOE, the central benzene ring makes a dihedral angle of 42.71 (7)° with the bromo­phenyl ring.

## Hirshfeld surface analysis   

**Figure 3:**
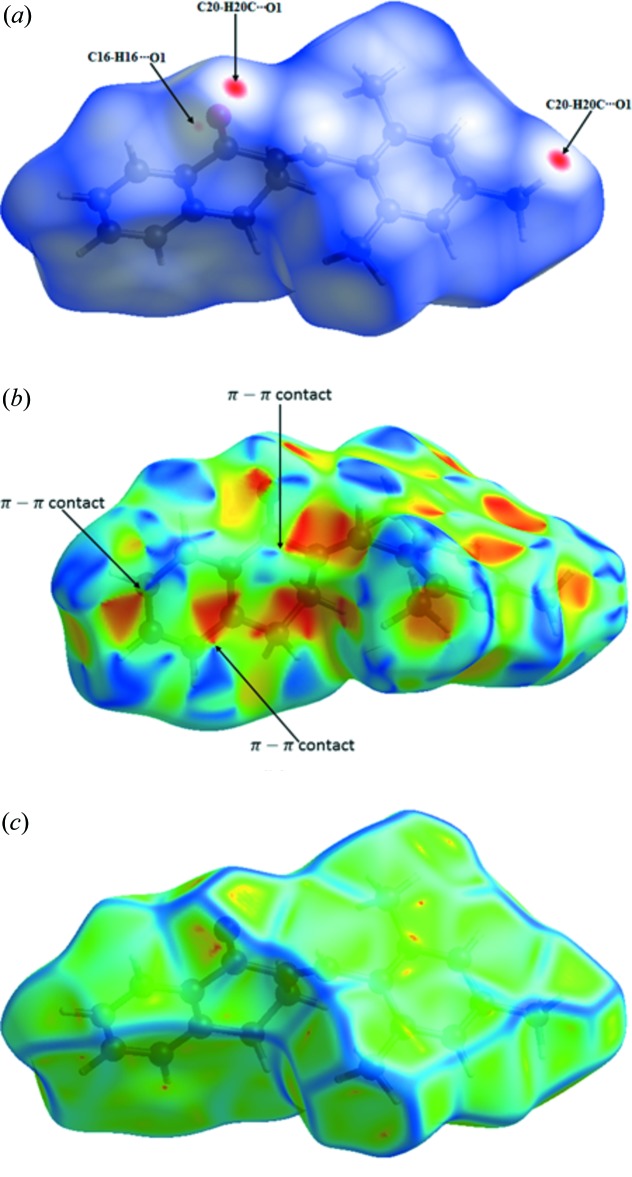
(*a*) *d_norm_* mapped on Hirshfeld surfaces for visualizing the inter­molecular inter­actions; (*b*) shape-index map and *(c)* curvedness map of the title compound.

**Figure 5:**
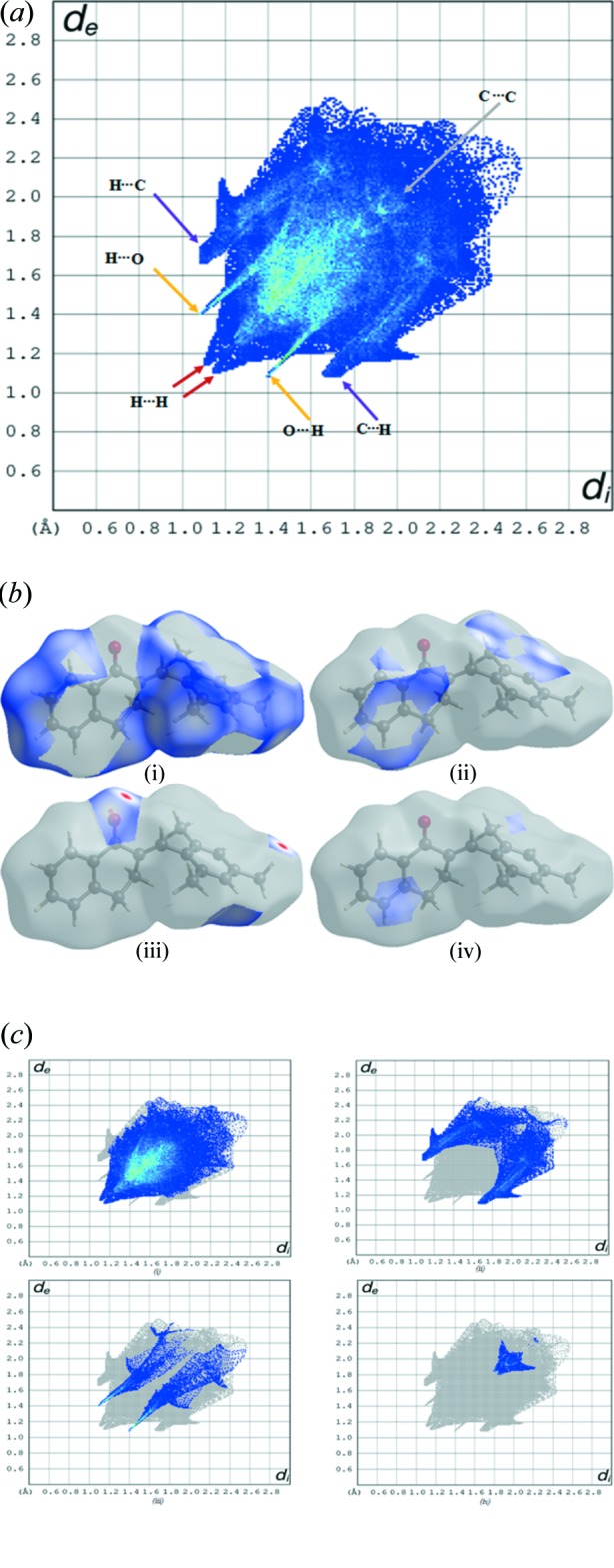
(*a*) The overall two-dimensional fingerprint plot and (*b*) Hirshfeld surface representations with the function *d*_norm_ plotted onto the surface for (i) H⋯H, (ii) H⋯C/C⋯H, (iii) H⋯O/O⋯H and (iv) C⋯C inter­actions. (*c*) The two-dimensional fingerprint plots for the title compound, delineated into (i) H⋯H, (ii) H⋯C/ C⋯H, (iii) H⋯O/O⋯H, (iv) C⋯C inter­actions.

A Hirshfeld surface analysis (Spackman & Jayatilaka, 2009 ▸) and the associated two-dimensional fingerprint plots (McKinnon *et al.*, 2007 ▸) were performed with *CrystalExplorer17* (Turner *et al.*, 2017 ▸), using standard surface resolution with the three-dimensional *d*_norm_ surfaces plotted over a fixed colour scale of −0.0870 (red) to 1.2944 (blue) a.u.. The three-dimensional *d*_norm_ surface of the title mol­ecule is illustrated in Fig. 3 ▸ *a* and 4 ▸. The pale-red spots symbolize short contacts and negative *d*_norm_ values on the surface correspond to the C—H⋯O inter­actions described above (Table 1 ▸). The overall two-dimensional fingerprint plot is illustrated in Fig. 5 ▸ *a*. The Hirshfeld surfaces mapped over *d*_norm_ are shown for the H⋯H, H⋯C/ C⋯H, H⋯O/O⋯H, C⋯C contacts (McKinnon *et al.*, 2007 ▸), and the two-dimensional fingerprint plots are shown in Fig. 5 ▸ *b* and 5*c*, respectively, associated with their relative contributions to the Hirshfeld surface. The largest contribution to the overall crystal packing is from H⋯H inter­actions (66.0%); H⋯H contacts are shown in the middle region 1.10 Å < (*d*_i_ + *d*_e_) < 1.18 Å. H⋯C/C⋯H contacts contribute 22.3% to the Hirshfeld surface, resulting in two pairs of characteristic wings in the fingerprint plot. The pair of tips appears at 1.10 Å < (*d*_i_ + *d*_e_) < 1.65 Å. H⋯O/O⋯H contacts make a 9.3% contribution to the Hirshfeld surface. The contacts are represented by a pair of sharp spikes in the region 1.05 Å < (*d*_i_ + *d*_e_) < 1.4 Å in the fingerprint plot. The C⋯C contacts are a measure of π–π stacking inter­actions and contribute 2.4% to the Hirshfeld surface. They appear as an arrow-shaped distribution at 1.80 Å < (*d*_i_ + *d*_e_) < 2.0 Å.

The shape-index map of the title mol­ecule (Fig. 3 ▸ *b*) was generated in the ranges −1 to 1 Å. The convex blue regions symbolize hydrogen-donor groups and concave red regions symbolize hydrogen-acceptor groups. π–π inter­actions on the shape-index map are indicated by adjacent red and blue triangles. As can be seen in Fig. 3 ▸ *b*, there are π–π inter­actions present between adjacent mol­ecules in the title complex.

The curvedness map of the title compound (Fig. 3 ▸ *c*) was generated in the range −4 to 0.4 Å. The large green regions represent a relatively flat (*i.e*. planar) surface area, while the blue regions demonstrate areas of curvature. The presence of π–π stacking inter­actions is also evident as flat regions around the rings on the Hirshfeld surface plotted over curvedness.

## Synthesis and crystallization   

2,4,6-Tri­methyl­benzyl­idene­tetra­lone was prepared according to a literature protocol (Kumar *et al.*, 2017 ▸). 10 ml of a NaOH solution (40%_wt_) was slowly added to a mixture of tetra­lone (1 mmol) and 2,4,6-tri­methyl­benzaldehyde (1 mmol) in ethanol (10 ml) at room temperature and stirred overnight. Then ice-cold water was added to the reaction mixture. The resulting precipitate was filtered off and dried *in vacuo*. The compound was purified by crystallization from ethanol, resulting in colourless prismatic crystals.

Yield 85%, m.p. 358 K; IR (ν, cm^−1^): 3060 (C—H, aromatic), 2920 (C—H, aliphatic), 1670 (C=O), 1620 (C=C, aromatic); ^1^H NMR (300 MHz, DMSO-*d*_6_, δ, ppm): 7.9 (1H, *d*, =C—H), 7.58 (1H, *s*, =C—H), 7.50 (1H, *t*, =C—H), 7.38 (1H,t, =C—H), 7.30 (1H, d, =C—H), 6.82 (2H, s, =C—H), 2.8 (2H, t, —CH_2_), 2.4 (2H, t, —CH_2_), 2.2 (3H, s,—CH_3_), 2.02 (6H, s, 2 CH_3_); ^13^C NMR (75 MHz, DMSO-*d*_6_, δ, ppm): 186.9, 144.5, 138.0, 137.2, 135.9, 135.6, 134.2, 133.5, 132.4, 129.3, 128.6, 128.0, 127.6, 28.9, 27.4, 21.3, 20.5. Analysis calculated for C_20_H_20_O: C, 86.92%; H, 7.29%; O, 5.79%. Found: C, 86.99%; H, 7.35%; O, 5.90%.

## Refinement details   

Crystal data, data collection and structure refinement details are summarized in Table 2 ▸. Hydrogen atoms were fixed geometrically and treated as riding, with C—H = 0.97 Å for methyl groups, 0.96 Å for methyl­ene groups, 0.93 Å for aromatic hydrogen atoms and 0.98 Å for methine groups, with *U*_iso_(H) = 1.2*U*_eq_(C) or 1.5*U*_eq_(C-meth­yl).

## Supplementary Material

Crystal structure: contains datablock(s) I. DOI: 10.1107/S2056989019006182/wm5495sup1.cif

Structure factors: contains datablock(s) I. DOI: 10.1107/S2056989019006182/wm5495Isup3.hkl

Supporting information file. DOI: 10.1107/S2056989019006182/wm5495Isup3.cml

CCDC reference: 1913649

Additional supporting information:  crystallographic information; 3D view; checkCIF report

## Figures and Tables

**Figure 4:**
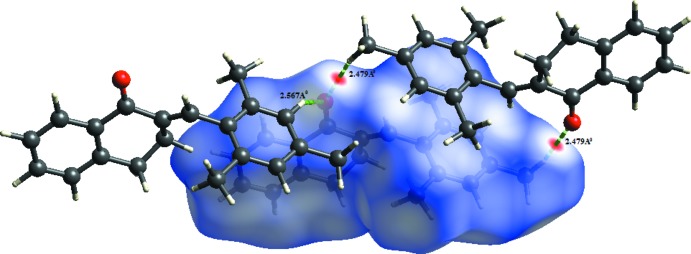
*d_norm_* mapped on Hirshfeld surfaces for visualizing the inter­molecular inter­actions.

**Table 1:** Hydrogen-bond geometry (Å, °) *Cg*2 is the centroid of the C2–C7 ring.

*D*—H⋯*A*	*D*—H	H⋯*A*	*D*⋯*A*	*D*—H⋯*A*
C16—H16⋯O1^i^	0.93	2.69	3.493 (5)	145
C20—H20*C*⋯O1^ii^	0.96	2.60	3.535 (5)	165
C9—H9*A*⋯*Cg*2^iii^	0.97	2.90	3.865 (6)	175

Symmetry codes: (i) 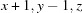; (ii) 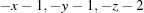; (iii) 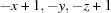.

**Table 2:** Experimental details

Crystal data	
Chemical formula	C_20_H_20_O
*M*_r_	276.36
Crystal system, space group	Triclinic, *P* 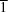
Temperature (K)	293
*a*, *b*, *c* (Å)	8.728 (2), 8.757 (2), 12.094 (3)
α, β, γ (°)	77.768 (19), 80.822 (19), 61.929 (18)
*V* (Å^3^)	795.2 (4)
*Z*	2
Radiation type	Mo *K*α
μ (mm^−1^)	0.07
Crystal size (mm)	0.64 × 0.51 × 0.33

Data collection	
Diffractometer	Stoe IPDS 2
Absorption correction	Integration (*X-RED32*; Stoe & Cie, 2002 ▸)
*T*_min_, *T*_max_	0.956, 0.982
No. of measured, independent and observed [*I* > 2σ(*I*)] reflections	8143, 2726, 1102
*R*_int_	0.088
(sin θ/λ)_max_ (Å^−1^)	0.595

Refinement	
*R*[*F* ^2^ > 2σ(*F* ^2^)], *wR*(*F* ^2^), *S*	0.061, 0.155, 0.91
No. of reflections	2726
No. of parameters	194
H-atom treatment	H-atom parameters constrained
Δρ_max_, Δρ_min_ (e Å^−3^)	0.25, −0.14

Computer programs: *X-AREA* and *X-RED* (Stoe & Cie, 2002 ▸), *SHELXT2017* (Sheldrick, 2015*a* ▸), *SHELXL2018* (Sheldrick, 2015*b* ▸), *Mercury* (Macrae *et al.*, 2008 ▸), *WinGX* (Farrugia, 2012 ▸), *PLATON* (Spek, 2009 ▸) and *publCIF* (Westrip, 2010 ▸).

## supplementary crystallographic information

### Crystal data

**Table:**

C_20_H_20_O	*Z* = 2
*M**_r_* = 276.36	*F*(000) = 296
Triclinic, *P*1	*D*_x_ = 1.154 Mg m^−^^3^
*a* = 8.728 (2) Å	Mo *K*α radiation, λ = 0.71073 Å
*b* = 8.757 (2) Å	Cell parameters from 12610 reflections
*c* = 12.094 (3) Å	θ = 2.7–30.2°
α = 77.768 (19)°	µ = 0.07 mm^−^^1^
β = 80.822 (19)°	*T* = 293 K
γ = 61.929 (18)°	Prism, colorless
*V* = 795.2 (4) Å^3^	0.64 × 0.51 × 0.33 mm

### Data collection

**Table:**

Stoe IPDS 2 diffractometer	1102 reflections with *I* > 2σ(*I*)
Detector resolution: 6.67 pixels mm^-1^	*R*_int_ = 0.088
rotation method scans	θ_max_ = 25.0°, θ_min_ = 2.7°
Absorption correction: integration (X-RED32; Stoe & Cie, 2002)	*h* = −10→10
*T*_min_ = 0.956, *T*_max_ = 0.982	*k* = −10→10
8143 measured reflections	*l* = −14→14
2726 independent reflections	

### Refinement

**Table:**

Refinement on *F*^2^	Hydrogen site location: inferred from neighbouring sites
Least-squares matrix: full	H-atom parameters constrained
*R*[*F*^2^ > 2σ(*F*^2^)] = 0.061	*w* = 1/[σ^2^(*F*_o_^2^) + (0.0601*P*)^2^] where *P* = (*F*_o_^2^ + 2*F*_c_^2^)/3
*wR*(*F*^2^) = 0.155	(Δ/σ)_max_ < 0.001
*S* = 0.91	Δρ_max_ = 0.25 e Å^−^^3^
2726 reflections	Δρ_min_ = −0.14 e Å^−^^3^
194 parameters	Extinction correction: SHELXL2018 (Sheldrick, 2015b), Fc^*^=kFc[1+0.001xFc^2^λ^3^/sin(2θ)]^-1/4^
0 restraints	Extinction coefficient: 0.016 (4)

### Special details

**Table:**

Geometry. All esds (except the esd in the dihedral angle between two l.s. planes) are estimated using the full covariance matrix. The cell esds are taken into account individually in the estimation of esds in distances, angles and torsion angles; correlations between esds in cell parameters are only used when they are defined by crystal symmetry. An approximate (isotropic) treatment of cell esds is used for estimating esds involving l.s. planes.

### Fractional atomic coordinates and isotropic or equivalent isotropic displacement parameters (Å^2^)

**Table:**

	*x*	*y*	*z*	*U*_iso_*/*U*_eq_	
O1	−1.0524 (3)	−0.2385 (4)	−0.7862 (2)	0.0977 (9)	
C12	−0.4930 (4)	−0.5186 (5)	−0.8354 (3)	0.0635 (9)	
C2	−1.0387 (4)	−0.2214 (4)	−0.5971 (3)	0.0639 (9)	
C17	−0.4278 (4)	−0.6907 (5)	−0.8516 (3)	0.0705 (10)	
C1	−0.9638 (4)	−0.2867 (5)	−0.7056 (3)	0.0715 (10)	
C10	−0.7746 (4)	−0.4083 (4)	−0.7133 (3)	0.0695 (10)	
C13	−0.3805 (5)	−0.4437 (5)	−0.8456 (3)	0.0744 (10)	
C11	−0.6837 (4)	−0.4120 (4)	−0.8119 (3)	0.0736 (11)	
H11	−0.745928	−0.339613	−0.873942	0.088*	
C15	−0.1364 (4)	−0.7151 (5)	−0.8870 (3)	0.0711 (10)	
C7	−0.9379 (4)	−0.2800 (5)	−0.5040 (3)	0.0743 (10)	
C16	−0.2502 (5)	−0.7860 (5)	−0.8766 (3)	0.0762 (11)	
H16	−0.206551	−0.902035	−0.886559	0.091*	
C14	−0.2038 (5)	−0.5443 (5)	−0.8704 (3)	0.0798 (11)	
H14	−0.128920	−0.494447	−0.875919	0.096*	
C3	−1.2113 (4)	−0.0905 (5)	−0.5883 (3)	0.0792 (11)	
H3	−1.280000	−0.051417	−0.649452	0.095*	
C9	−0.7027 (5)	−0.5193 (6)	−0.6044 (3)	0.1051 (15)	
H9A	−0.745452	−0.606334	−0.583241	0.126*	
H9B	−0.576855	−0.580756	−0.615353	0.126*	
C8	−0.7503 (4)	−0.4167 (5)	−0.5111 (3)	0.0951 (13)	
H8A	−0.676588	−0.358974	−0.520287	0.114*	
H8B	−0.726257	−0.496759	−0.439895	0.114*	
C4	−1.2801 (5)	−0.0192 (5)	−0.4895 (4)	0.0909 (13)	
H4	−1.394381	0.067973	−0.484398	0.109*	
C6	−1.0115 (5)	−0.2062 (6)	−0.4058 (3)	0.0942 (13)	
H6	−0.945571	−0.244732	−0.343260	0.113*	
C5	−1.1799 (6)	−0.0771 (6)	−0.4000 (4)	0.0988 (14)	
H5	−1.226312	−0.028553	−0.333787	0.119*	
C20	0.0576 (4)	−0.8225 (6)	−0.9157 (3)	0.1039 (15)	
H20A	0.094884	−0.940932	−0.877619	0.156*	
H20B	0.121224	−0.772547	−0.891430	0.156*	
H20C	0.078722	−0.821651	−0.996162	0.156*	
C18	−0.5449 (5)	−0.7765 (5)	−0.8436 (4)	0.1077 (15)	
H18A	−0.589023	−0.792507	−0.766309	0.162*	
H18B	−0.479794	−0.888378	−0.868695	0.162*	
H18C	−0.640231	−0.703345	−0.890605	0.162*	
C19	−0.4477 (5)	−0.2554 (5)	−0.8281 (4)	0.1150 (16)	
H19A	−0.531484	−0.178397	−0.882365	0.173*	
H19B	−0.352349	−0.226633	−0.838182	0.173*	
H19C	−0.501571	−0.241994	−0.752796	0.173*	

### Atomic displacement parameters (Å^2^)

**Table:**

	*U*^11^	*U*^22^	*U*^33^	*U*^12^	*U*^13^	*U*^23^
O1	0.0782 (16)	0.112 (2)	0.088 (2)	−0.0233 (15)	−0.0269 (14)	−0.0175 (16)
C12	0.069 (2)	0.057 (2)	0.060 (2)	−0.0271 (19)	−0.0034 (16)	−0.0044 (18)
C2	0.061 (2)	0.066 (2)	0.066 (2)	−0.0311 (19)	−0.0055 (18)	−0.0045 (19)
C17	0.079 (2)	0.061 (3)	0.073 (3)	−0.032 (2)	−0.0094 (18)	−0.0086 (19)
C1	0.073 (2)	0.071 (3)	0.069 (3)	−0.031 (2)	−0.019 (2)	−0.001 (2)
C10	0.062 (2)	0.070 (3)	0.062 (2)	−0.0218 (19)	−0.0091 (18)	0.0017 (19)
C13	0.082 (3)	0.058 (3)	0.081 (3)	−0.032 (2)	0.0010 (19)	−0.014 (2)
C11	0.072 (2)	0.072 (3)	0.072 (3)	−0.029 (2)	−0.0174 (19)	−0.001 (2)
C15	0.077 (2)	0.070 (3)	0.058 (2)	−0.026 (2)	−0.0030 (18)	−0.012 (2)
C7	0.071 (2)	0.084 (3)	0.067 (2)	−0.037 (2)	−0.005 (2)	−0.006 (2)
C16	0.095 (3)	0.059 (3)	0.069 (2)	−0.028 (2)	−0.010 (2)	−0.0122 (19)
C14	0.082 (3)	0.081 (3)	0.085 (3)	−0.045 (2)	0.0018 (19)	−0.015 (2)
C3	0.069 (2)	0.077 (3)	0.091 (3)	−0.032 (2)	−0.013 (2)	−0.008 (2)
C9	0.092 (3)	0.101 (3)	0.079 (3)	−0.013 (2)	−0.015 (2)	0.003 (3)
C8	0.080 (3)	0.099 (3)	0.081 (3)	−0.018 (2)	−0.024 (2)	−0.003 (3)
C4	0.076 (3)	0.085 (3)	0.102 (3)	−0.030 (2)	0.008 (3)	−0.022 (3)
C6	0.095 (3)	0.109 (3)	0.072 (3)	−0.040 (3)	−0.010 (2)	−0.012 (3)
C5	0.101 (3)	0.107 (4)	0.085 (3)	−0.045 (3)	0.008 (3)	−0.025 (3)
C20	0.076 (3)	0.113 (4)	0.098 (3)	−0.020 (2)	0.003 (2)	−0.030 (3)
C18	0.114 (3)	0.093 (3)	0.141 (4)	−0.065 (3)	−0.010 (3)	−0.023 (3)
C19	0.107 (3)	0.073 (3)	0.172 (5)	−0.044 (2)	0.007 (3)	−0.038 (3)

### Geometric parameters (Å, º)

**Table:**

O1—C1	1.218 (4)	C3—C4	1.383 (5)
C12—C17	1.384 (4)	C3—H3	0.9300
C12—C13	1.393 (4)	C9—C8	1.477 (5)
C12—C11	1.491 (4)	C9—H9A	0.9700
C2—C7	1.396 (5)	C9—H9B	0.9700
C2—C3	1.404 (4)	C8—H8A	0.9700
C2—C1	1.473 (4)	C8—H8B	0.9700
C17—C16	1.390 (4)	C4—C5	1.359 (5)
C17—C18	1.510 (5)	C4—H4	0.9300
C1—C10	1.486 (4)	C6—C5	1.371 (5)
C10—C11	1.319 (4)	C6—H6	0.9300
C10—C9	1.490 (5)	C5—H5	0.9300
C13—C14	1.389 (4)	C20—H20A	0.9600
C13—C19	1.519 (5)	C20—H20B	0.9600
C11—H11	0.9300	C20—H20C	0.9600
C15—C14	1.373 (5)	C18—H18A	0.9600
C15—C16	1.377 (5)	C18—H18B	0.9600
C15—C20	1.524 (4)	C18—H18C	0.9600
C7—C6	1.390 (5)	C19—H19A	0.9600
C7—C8	1.508 (5)	C19—H19B	0.9600
C16—H16	0.9300	C19—H19C	0.9600
C14—H14	0.9300		
			
C17—C12—C13	119.7 (3)	C10—C9—H9A	109.0
C17—C12—C11	120.0 (3)	C8—C9—H9B	109.0
C13—C12—C11	120.3 (3)	C10—C9—H9B	109.0
C7—C2—C3	119.0 (3)	H9A—C9—H9B	107.8
C7—C2—C1	121.2 (3)	C9—C8—C7	114.6 (4)
C3—C2—C1	119.8 (4)	C9—C8—H8A	108.6
C12—C17—C16	119.0 (3)	C7—C8—H8A	108.6
C12—C17—C18	121.7 (3)	C9—C8—H8B	108.6
C16—C17—C18	119.3 (4)	C7—C8—H8B	108.6
O1—C1—C2	121.3 (3)	H8A—C8—H8B	107.6
O1—C1—C10	121.8 (3)	C5—C4—C3	119.6 (4)
C2—C1—C10	116.9 (4)	C5—C4—H4	120.2
C11—C10—C1	119.9 (3)	C3—C4—H4	120.2
C11—C10—C9	125.0 (3)	C5—C6—C7	120.9 (4)
C1—C10—C9	115.1 (3)	C5—C6—H6	119.6
C14—C13—C12	119.3 (3)	C7—C6—H6	119.6
C14—C13—C19	119.7 (4)	C4—C5—C6	121.0 (4)
C12—C13—C19	121.1 (3)	C4—C5—H5	119.5
C10—C11—C12	127.7 (3)	C6—C5—H5	119.5
C10—C11—H11	116.2	C15—C20—H20A	109.5
C12—C11—H11	116.2	C15—C20—H20B	109.5
C14—C15—C16	117.6 (3)	H20A—C20—H20B	109.5
C14—C15—C20	121.2 (4)	C15—C20—H20C	109.5
C16—C15—C20	121.2 (4)	H20A—C20—H20C	109.5
C6—C7—C2	118.9 (3)	H20B—C20—H20C	109.5
C6—C7—C8	120.4 (4)	C17—C18—H18A	109.5
C2—C7—C8	120.6 (3)	C17—C18—H18B	109.5
C15—C16—C17	122.4 (4)	H18A—C18—H18B	109.5
C15—C16—H16	118.8	C17—C18—H18C	109.5
C17—C16—H16	118.8	H18A—C18—H18C	109.5
C15—C14—C13	122.0 (4)	H18B—C18—H18C	109.5
C15—C14—H14	119.0	C13—C19—H19A	109.5
C13—C14—H14	119.0	C13—C19—H19B	109.5
C4—C3—C2	120.6 (4)	H19A—C19—H19B	109.5
C4—C3—H3	119.7	C13—C19—H19C	109.5
C2—C3—H3	119.7	H19A—C19—H19C	109.5
C8—C9—C10	112.8 (4)	H19B—C19—H19C	109.5
C8—C9—H9A	109.0		
			
C13—C12—C17—C16	0.7 (5)	C3—C2—C7—C8	−178.2 (3)
C11—C12—C17—C16	178.2 (3)	C1—C2—C7—C8	−2.0 (5)
C13—C12—C17—C18	−178.9 (3)	C14—C15—C16—C17	0.8 (5)
C11—C12—C17—C18	−1.3 (5)	C20—C15—C16—C17	−179.4 (3)
C7—C2—C1—O1	178.4 (4)	C12—C17—C16—C15	−0.7 (5)
C3—C2—C1—O1	−5.3 (5)	C18—C17—C16—C15	178.9 (3)
C7—C2—C1—C10	−3.8 (5)	C16—C15—C14—C13	−1.0 (5)
C3—C2—C1—C10	172.5 (3)	C20—C15—C14—C13	179.2 (3)
O1—C1—C10—C11	27.7 (6)	C12—C13—C14—C15	1.0 (5)
C2—C1—C10—C11	−150.1 (3)	C19—C13—C14—C15	−179.9 (4)
O1—C1—C10—C9	−152.2 (4)	C7—C2—C3—C4	0.7 (5)
C2—C1—C10—C9	30.0 (5)	C1—C2—C3—C4	−175.6 (3)
C17—C12—C13—C14	−0.8 (5)	C11—C10—C9—C8	129.8 (4)
C11—C12—C13—C14	−178.4 (3)	C1—C10—C9—C8	−50.3 (5)
C17—C12—C13—C19	−179.9 (4)	C10—C9—C8—C7	43.9 (5)
C11—C12—C13—C19	2.6 (5)	C6—C7—C8—C9	163.5 (4)
C1—C10—C11—C12	177.2 (3)	C2—C7—C8—C9	−18.7 (6)
C9—C10—C11—C12	−2.9 (7)	C2—C3—C4—C5	−0.4 (6)
C17—C12—C11—C10	83.3 (5)	C2—C7—C6—C5	−0.3 (6)
C13—C12—C11—C10	−99.2 (5)	C8—C7—C6—C5	177.5 (4)
C3—C2—C7—C6	−0.4 (5)	C3—C4—C5—C6	−0.3 (7)
C1—C2—C7—C6	175.9 (3)	C7—C6—C5—C4	0.7 (7)

### Hydrogen-bond geometry (Å, º)

*Cg*2 is the centroid of the C2–C7 ring.

**Table:**

*D*—H···*A*	*D*—H	H···*A*	*D*···*A*	*D*—H···*A*
C16—H16···O1^i^	0.93	2.69	3.493 (5)	145
C20—H20*C*···O1^ii^	0.96	2.60	3.535 (5)	165
C9—H9*A*···*Cg*2^iii^	0.97	2.90	3.865 (6)	175

Symmetry codes: (i) *x*+1, *y*−1, *z*; (ii) −*x*−1, −*y*−1, −*z*−2; (iii) −*x*+1, −*y*, −*z*+1.
